# Family socioeconomic status and attention-deficit/hyperactivity disorder symptoms in preschool children: a cross-sectional study of 21,366 parent–child dyads in Yan’an, western China

**DOI:** 10.3389/fpsyt.2026.1943855

**Published:** 2026-09-28

**Authors:** Jiali Du, Man Bai, Jieqiong Liu, Jie Mi, Yan Wang

**Affiliations:** 1 School of Educational Sciences, Yan’an University, Yan’an, Shaanxi, China; 2 Shaanxi Pharmaceutical Information Center, Shaanxi Institute of Chinese Materia Medica, Xianyang, Shaanxi, China; 3 Department of Pharmacy, Second Affiliated Hospital of Shaanxi University of Chinese Medicine, Xianyang, Shaanxi, China; 4 Department of Pharmacy, Xi’an Children’s Hospital, Affiliated Children’s Hospital of Xi’an Jiaotong University, National Regional Children’s Medical Center (Northwest), Xi’an, Shaanxi, China; 5 Department of Pediatrics, Xi’an No. 9 Hospital, Xi’an, Shaanxi, China

**Keywords:** attention-deficit/hyperactivity disorder, china, cross-sectional study, preschool children, socioeconomic status

## Abstract

**Background:**

Socioeconomic status (SES) is a well-established determinant of child mental health, yet evidence from large preschool populations in western China remains scarce. This study examined the association between family SES and attention-deficit/hyperactivity disorder (ADHD) symptoms in preschool children.

**Methods:**

We conducted a cross-sectional survey among 21,366 parent–child dyads recruited from 189 public kindergartens in a western Chinese city using stratified cluster sampling. A composite family SES index was derived from parental occupation, education, and household income through principal component analysis. Child ADHD symptoms were measured using the Strengths and Difficulties Questionnaire (SDQ) hyperactivity/inattention subscale. Survey-weighted linear and logistic regression models adjusted for child age, sex, preterm birth, parental smoking and alcohol use, respondent role, and respondent age, with cluster-robust standard errors accounting for kindergarten-level clustering.

**Results:**

Higher family SES was significantly associated with lower ADHD symptom scores. In the fully adjusted linear model, each 1-SD increase in SES corresponded to a 0.17-point decrease in the SDQ hyperactivity/inattention score (95% CI: –0.18 to –0.15, P < 0.001). Logistic regression showed a 27% reduction in the odds of elevated ADHD symptoms per 1-SD increase in SES (OR = 0.73, 95% CI: 0.64 to 0.84, P < 0.001). A clear dose–response relationship emerged across SES tertiles (P for trend < 0.001). Stratified analyses demonstrated consistent associations across all subgroups, with no significant effect modification after Bonferroni correction. Multiple imputation and restricted cubic spline analyses confirmed the robustness of these findings.

**Conclusions:**

Higher family SES is associated with lower ADHD symptom levels in preschool children from western China, with a clear dose-response relationship. These findings underscore the importance of socioeconomic resources in early childhood mental health and may inform the development of targeted intervention strategies for low-SES families. Longitudinal studies are warranted to establish causality and elucidate underlying mechanisms.

## 1 Introduction

Attention-deficit/hyperactivity disorder (ADHD) is one of the most common neurodevelopmental disorders in childhood, affecting approximately 6.3% of children and adolescents in China (1). The disorder often persists into adolescence and adulthood and is associated with impairments in academic performance, social functioning, and quality of life. Given the early onset of ADHD symptoms and their potential long-term consequences, identifying modifiable risk factors during the preschool period is critical for early prevention and intervention.

Socioeconomic status (SES), typically measured by parental education, occupation, and family income, has been consistently identified as a key determinant of child mental health. A growing body of evidence has demonstrated that lower SES is associated with higher ADHD symptom levels and greater risk of ADHD diagnosis across diverse populations. In preschool-aged children, SES was negatively related to ADHD traits and emotional-behavioral profiles (2), and the association between SES and ADHD symptoms was found to be partially mediated by executive function, particularly working memory and inhibitory ability (3). Research demonstrated an interaction between family income and parental history of ADHD: among children without a parental history, low-income children had 6.2 times the odds of ADHD compared to high-income children, suggesting that SES may be particularly important in the absence of genetic predisposition (4). Additionally, the association between social determinants of health and ADHD symptoms was found to be fully mediated by caregiver well-being, highlighting the role of family environment in this pathway (5).

Population-based studies have further documented socioeconomic disparities in ADHD prevalence and treatment. In a Spanish cohort of over 600,000 individuals aged 1–30 years, the low SES group had a significantly higher incidence of ADHD and other mental disorders (6). In the United States, economic hardship, unsafe neighborhoods, and family structure were positively correlated with ADHD diagnosis (7), and sociodemographic disparities in diagnosis and treatment persist even after adjusting for symptom severity (8). In Australia, youth from lower socioeconomic regions were slightly more likely to be prescribed ADHD medication, while the highest socioeconomic decile showed the greatest increase in prescription rates over time (9). Similar patterns were observed in England, where socioeconomic deprivation was significantly associated with ADHD prescribing rates across regions (10). Moreover, higher SES has been associated with better global improvement in children with ADHD, suggesting that socioeconomic advantage may also influence treatment response and prognosis (11).

The mechanisms underlying the SES-ADHD association are multifaceted. Neuroimaging studies have shown that reduced subcortical volume in the cerebellum and caudate mediated the association between low SES and ADHD status (12), suggesting environmentally mediated changes in brain structure may explain elevated risk in low-SES families. Beyond neurobiological pathways, a causal pathway from SES to ADHD symptoms has been identified, partially mediated by asthma, with increased family income directly reducing ADHD symptoms (13). Mendelian randomization studies have further strengthened the causal evidence: genetically predicted higher education and family income conferred significantly lower odds of ADHD diagnosis, and the findings suggest that ADHD may be a direct and strong intelligence-independent consequence of socioeconomic factors rather than the reverse (14).

Despite the growing evidence, several important gaps remain. Most existing studies have been conducted in Western populations or in eastern coastal cities of China, with limited evidence from western regions where socioeconomic conditions differ substantially. Furthermore, many prior studies have used single indicators of SES rather than comprehensive indices incorporating both parents’ education and occupation. To address these gaps, we conducted a large cross-sectional survey of 21,366 parent–child dyads recruited from 189 public kindergartens in a city of western China. We constructed a comprehensive family SES index via principal component analysis incorporating paternal and maternal occupation and education as well as household income. We aimed to examine the association between family SES and preschool children’s ADHD symptoms, assess the dose–response relationship, and test whether this association was consistent across key subgroups. We hypothesized that higher family SES would be associated with lower ADHD symptom levels, and that this association would be robust across different population subgroups.

## 2 Methods

### 2.1 Study design and population

This cross-sectional survey was conducted in a city in western China. Using a stratified cluster sampling design, we recruited children aged 3–6 years from 189 public kindergartens, with strata defined by the city’s 13 administrative districts. Within each district, kindergartens were randomly selected from the official roster, and the number of kindergartens was proportionally allocated based on each district’s share of the total child population. Data collection occurred between February 28 and March 5, 2025. Parents provided informed consent, and one parent per child completed the questionnaire. Of 28,000 distributed questionnaires, 25,017 were returned (89.3% overall questionnaire return rate). After excluding non-parental respondents (n = 2,408), outlying parental age (n = 517), and invalid child age (n = 726), the final analytic sample comprised 21,366 parent–child dyads. The study was approved by the Ethics Committee of the Affiliated Children’s Hospital of Xi’an Jiaotong University (Approval NO. 20250225-21).

### 2.2 Family SES

We measured family SES using three core dimensions: parental occupation, parental education, and annual household income.

Parental occupation. The responding parent and their spouse reported their occupations from twelve categories derived from the Chinese Standard Classification of Occupations. Following established protocols (15), each occupation category was assigned a score from 1 (lowest) to 10 (highest) based on a comprehensive assessment of occupational prestige in the contemporary Chinese context. This approach is consistent with the international framework for constructing socio-economic indices based on occupational status (16). Occupations such as civil servants received the highest score of 10, while housewives/husbands and retirees received 3 (15). The method of assigning socio-economic scores to occupations aligns with well-established approaches such as the Blishen and Hollingshead scales (17).

Parental education. The highest educational level attained by each parent was reported using an eight-level scale (from “incomplete primary school” to “doctoral degree”). To create a continuous measure, this was converted into years of formal schooling according to the standard Chinese education system (6 years for primary school, 3 years for junior high school, 3 years for senior high school, etc.), a widely adopted approach in Chinese longitudinal surveys and birth cohort studies (18). The conversion was as follows: incomplete primary school = 5 years; primary school graduate = 6 years; junior high school graduate = 9 years; senior high school graduate = 12 years; associate degree = 15 years; bachelor’s degree = 16 years; master’s degree = 18 years; and doctoral degree = 22 years.

Household income. Annual household income was reported across eight brackets (ranging from “<10,000 RMB” to “≥300,000 RMB”). For analysis, each bracket was assigned its midpoint value in thousands of RMB (e.g., “100,000–149,999 RMB” was coded as 125). For the lowest (<10,000 RMB) and highest (≥300,000 RMB) open-ended brackets, we assigned 5 and 350 (19), respectively, following the conventional approach for censored income categories in SES research. This midpoint coding approach is a widely used method for transforming categorical income variables into continuous measures in SES research, and has been successfully applied in recent studies using Chinese samples (19).

Construction of the family SES index. To construct a single, continuous family SES index, principal component analysis (PCA) was performed on five standardized variables: (1) paternal occupation score, (2) paternal education years, (3) maternal occupation score, (4) maternal education years, and (5) household income (midpoint). PCA is a well-established method for constructing SES indices (20). Similar SES indices based on PCA have been successfully applied in family SES relationship research using Chinese birth cohort data (21) and in maternal and child health research in rural China (22). The first principal component (PC1) accounted for 52.4% of the total variance. The loadings of the five variables on PC1 were –0.44 (paternal occupation), –0.51 (paternal education), –0.46 (maternal occupation), –0.51 (maternal education), and –0.29 (household income), confirming that all components contributed substantially to the SES dimension. All five original variables loaded negatively on PC1; therefore, to enhance interpretability (so that a higher score indicates a higher SES), the PC1 scores were multiplied by −1. The resulting standardized family SES index (mean = 0, SD = 1) was used as the primary independent variable in all regression analyses.

### 2.3 Child ADHD symptoms

Child ADHD symptoms were assessed using the hyperactivity/inattention subscale of the Strengths and Difficulties Questionnaire (SDQ), a validated 25-item screening tool for emotional and behavioral problems in children (23–26). This subscale comprises five items rated on a 3-point scale (0 = not true, 1 = somewhat true, 2 = certainly true), yielding a total score from 0 to 10, with higher scores indicating greater symptom severity. Both the continuous total score and a binary indicator (score >6) were used as outcomes, consistent with established Chinese SDQ cutoff values (24, 27, 28), and prior epidemiological studies using this subscale for ADHD screening (26, 29). The Chinese version of the SDQ has demonstrated acceptable reliability and validity for assessing ADHD-related symptoms in children aged 3–17 years (24, 29). In the present sample, Cronbach’s α for the subscale was 0.70, indicating satisfactory internal consistency. It is important to note that the SDQ is a screening instrument rather than a diagnostic tool, therefore, our findings reflect ADHD symptoms as measured by this subscale, rather than clinically diagnosed ADHD.

### 2.4 Covariates

The following covariates were included in the fully adjusted models: child age (months, continuous), child sex (boy vs. girl), preterm birth (yes/no; defined as gestational age <37 weeks), maternal smoking (yes/no), paternal smoking (yes/no), maternal alcohol use (yes/no), paternal alcohol use (yes/no), respondent role (mother vs. father; to account for potential reporting bias), and respondent age (years, continuous). All covariates were collected via the parent-reported questionnaire.

### 2.5 Statistical analysis

All analyses were performed using R version 4.3.1. Given the stratified cluster sampling design with kindergartens as the primary sampling units, we adjusted for within-kindergarten correlation by using cluster-robust standard errors in all regression analyses. The family SES index was constructed as described in Section 2.2, and used as the main exposure variable. All regression models (linear for continuous outcome and logistic for binary outcome) adjusted for the same set of covariates: child age (months), child sex (boy vs. girl), preterm birth (yes/no), maternal smoking, paternal smoking, maternal alcohol use, paternal alcohol use, respondent role (mother vs. father), and respondent age (years). Results are presented as β (95% CI) for linear regression and OR (95% CI) for logistic regression.

Using this fully adjusted specification, we performed: (1) primary analysis with SES as a continuous variable (Model 1 unadjusted, Model 2 adjusted); (2) dose-response analysis by categorizing the continuous SES index into tertiles (low, middle, high) based on the 33.3rd and 66.6th percentiles of its distribution in the analytical sample, with a trend test performed by treating the tertiles as an ordinal variable (1, 2, 3) to evaluate linear trends across the three groups; and (3) stratified analyses by child age group (<4, ≥4 to <5, ≥5 years), child sex, preterm birth, respondent role, respondent age (<40 vs. ≥40 years), parental smoking, and parental alcohol use. For stratification, effect modification was tested by adding a cross-product term in the overall sample. Interaction P-values were derived from Wald tests for binary strata or from Type 3 ANOVA for multicategory strata, and Bonferroni correction was applied (α = 0.05/9 = 0.0056) to control the family-wise error rate.

Several sensitivity analyses were conducted to assess robustness. Multiple imputation for missing data was performed using the MICE package (5 imputations, predictive mean matching) on the full sample of 25,017 participants. After imputation, the same PCA-based SES index was reconstructed and the fully adjusted models were refitted, with results pooled via Rubin’s rules. Non-linearity was explored using restricted cubic splines (RCS) with 4 knots (the rms package for ordinary least squares linear regression), with the non-linear component tested by Wald statistic. Model diagnostics included Q-Q plots and residual-vs-fitted plots (linear regression), and AUC and Brier score (logistic regression). Multicollinearity was assessed using variance inflation factors (VIF) for all predictors in the fully adjusted linear model (VIF < 5 indicates negligible multicollinearity with no statistical concern). All P-values are two-sided, with statistical significance set at P < 0.05, except for Bonferroni-corrected analyses where the threshold was 0.0056 (0.05/9).

## 3 Results

### 3.1 Participant characteristics

Among the 21,366 parent–child dyads, the mean child age was 4.82 ± 0.89 years and 51.8% were boys. The mean respondent age was 34.75 ± 4.56 years and 76.1% of the respondents were mothers. **Table 1** presents the baseline characteristics stratified by family SES tertiles. Compared with families in the high SES group, those in the low SES group had older children (4.87 ± 0.89 vs. 4.78 ± 0.88 years, P<0.001), a higher proportion of maternal respondents (80% vs. 69%, P<0.001), higher rates of maternal smoking (1.5% vs. 0.5%, P<0.001) and paternal smoking (67% vs. 50%, P<0.001), and a higher prevalence of maternal alcohol use (4.8% vs. 2.9%, P<0.001). Educational attainment, employment status, and annual household income all showed strong relationships across SES tertiles (all P<0.001), confirming the validity of the SES classification. No significant differences were observed in child gender across groups (P = 0.7).

**Table 1:** Baseline characteristics by family socioeconomic status (SES) tertiles.

Characteristic	Overall N = 21,366	Low-SES N = 7,122	Middle-SES N = 7,122	High-SES N = 7,122	P-value
Child age (years)	4.82 (0.89)	4.87 (0.89)	4.80 (0.89)	4.78 (0.88)	<0.001
Child gender					0.7
Girl	10,304 (48%)	3,448 (48%)	3,405 (48%)	3,451 (48%)	
Boy	11,062 (52%)	3,674 (52%)	3,717 (52%)	3,671 (52%)	
Preterm birth	983 (4.6%)	356 (5.0%)	309 (4.3%)	318 (4.5%)	0.14
Respondent role					<0.001
Mother	16,258 (76%)	5,722 (80%)	5,609 (79%)	4,927 (69%)	
Father	5,108 (24%)	1,400 (20%)	1,513 (21%)	2,195 (31%)	
Respondent age (years)	34.75 (4.56)	34.56 (5.13)	34.12 (4.23)	35.58 (4.14)	<0.001
Maternal smoking	201 (0.9%)	110 (1.5%)	58 (0.8%)	33 (0.5%)	<0.001
Paternal smoking	12,692 (59%)	4,778 (67%)	4,378 (61%)	3,536 (50%)	<0.001
Maternal alcohol use	808 (3.8%)	341 (4.8%)	259 (3.6%)	208 (2.9%)	<0.001
Paternal alcohol use	9,615 (45%)	3,214 (45%)	3,174 (45%)	3,227 (45%)	0.6
Education level (respondent)					<0.001
≤ Junior high school	5,741 (27%)	5,079 (71%)	642 (9.0%)	20 (0.3%)	
High school & junior college	9,476 (44%)	2,003 (28%)	5,242 (74%)	2,231 (31%)	
≥ Undergraduate	6,149 (29%)	40 (0.6%)	1,238 (17%)	4,871 (68%)	
Occupation (respondent)					<0.001
Not working	5,724 (27%)	3,709 (52%)	1,764 (25%)	251 (3.5%)	
Working	15,642 (73%)	3,413 (48%)	5,358 (75%)	6,871 (96%)	
Marital status					<0.001
Married/Living with partner	20,761 (97%)	6,765 (95%)	6,957 (98%)	7,039 (99%)	
Others	605 (2.8%)	357 (5.0%)	165 (2.3%)	83 (1.2%)	
Annual family income (¥ thousands)					<0.001
< 30	8,619 (40%)	4,375 (61%)	2,706 (38%)	1,538 (22%)	
30-100	8,619 (40%)	2,460 (35%)	3,211 (45%)	2,948 (41%)	
≥ 100	4,128 (19%)	287 (4.0%)	1,205 (17%)	2,636 (37%)	
ADHD symptom score	2.23 (1.60)	2.53 (1.64)	2.18 (1.56)	1.99 (1.57)	<0.001

Values are mean (SD) or n (%). P-values from ANOVA for continuous variables and Rao-Scott chi-square tests for categorical variables, accounting for clustering by kindergarten. SES tertiles based on the continuous family SES index (reversed PC1). ADHD symptoms score was derived from the SDQ hyperactivity/inattention subscale.

### 3.2 Association between family SES and child ADHD symptoms

Higher family SES was significantly associated with lower ADHD symptom scores in both linear and logistic regression models after full adjustment for child age, child sex, preterm birth, parental smoking and alcohol use, respondent role, and respondent age (**Table 2**). In the fully adjusted linear model, each 1-SD increase in SES corresponded to a 0.17-point decrease in the SDQ hyperactivity/inattention score (95% CI: –0.18 to –0.15, P < 0.001). In the logistic regression using the binary outcome, higher SES was associated with a 27% reduction in the odds of elevated ADHD symptoms per 1-SD increase (OR = 0.73, 95% CI: 0.64 to 0.84, P < 0.001). Variance inflation factors for all predictors in the fully adjusted model were below 1.2, indicating no multicollinearity (**Supplementary Table 1**).

**Table 2:** Associations between family SES and child ADHD symptoms.

Outcome	Model 1 (unadjusted)	Model 2 (adjusted)	P value
ADHD symptom score (continuous)	-0.15 (-0.16, -0.14)	-0.17 (-0.18, -0.15)	<0.001
Elevated ADHD symptoms (binary)	0.72 (0.63, 0.82)	0.73 (0.64, 0.84)	<0.001

Model 1: unadjusted. Model 2 adjusted for child age (months), child sex (boy vs. girl), preterm birth (yes/no), maternal smoking, paternal smoking, maternal alcohol use, paternal alcohol use, respondent role (mother vs. father), and respondent age (years). All models accounted for clustering by kindergarten using cluster-robust standard errors. SES is the continuous family SES index (reversed PC1, mean 0, SD 1). β: change in ADHD symptom score (0–10) per 1-SD increase in SES. OR: odds of ADHD (score >6) per 1-SD increase in SES.

### 3.3 Dose–response and nonlinearity assessment

When SES was categorized into tertiles, a clear dose–response relationship emerged (**Table 3**). Compared with children from low-SES families (reference), those from middle-SES families had a 0.35-point lower ADHD symptom score (95% CI: –0.40 to –0.31), and those from high-SES families had a 0.59-point lower score (95% CI: –0.65 to –0.54). For the binary outcome, middle and high SES were associated with 62% (OR = 0.38, 95% CI: 0.24 to 0.61) and 66% (OR = 0.34, 95% CI: 0.21 to 0.55) reductions in the odds of elevated ADHD symptoms, respectively. The P for trend was <0.001 in both models, supporting a monotonic inverse association. Restricted cubic spline analysis revealed a statistically significant nonlinear component (P for nonlinearity <0.001). However, the RCS curve demonstrated a monotonic decreasing pattern across the entire SES spectrum, with a slightly steeper decline in the lower SES range and a modest attenuation at higher SES levels (**Supplementary Figure 1**). Although the nonlinear term reached statistical significance, the magnitude of deviation from linearity was small across the full SES distribution, and subgroup stratified analyses yielded consistent inverse linear associations. Given the large sample size driving the statistical significance of minor nonlinear departures, the linear SES index was retained as the primary exposure for parsimony and interpretability.

**Table 3:** Association between SES tertiles and child ADHD symptoms (continuous and binary outcomes).

SES group	Continuous	Binary
Low SES (ref)	Ref	Ref
Middle SES	-0.35 (-0.40, -0.31)	0.38 (0.24, 0.61)
High SES	-0.59 (-0.65, -0.54)	0.34 (0.21, 0.55)
P for trend	<0.001	<0.001

Models adjusted for child age, child sex, preterm birth, maternal smoking, paternal smoking, maternal alcohol use, paternal alcohol use, respondent role, and respondent age. All models accounted for clustering by kindergarten. P for trend derived from treating SES tertile as an ordinal variable (1,2,3). Continuous outcome: linear regression; binary outcome: logistic regression.

### 3.4 Stratified and interaction analyses

Stratified analyses yielded negative β estimates across all subgroups. The 95% CIs excluded the null value for nearly all subgroups, except the small maternal smoking subgroup (n=201) with a confidence interval crossing zero (**Table 4**). After Bonferroni correction for nine comparisons (α = 0.0056), none of the interaction terms reached statistical significance.

**Table 4:** Stratified analysis of the association between family SES and child ADHD symptoms.

Stratified variable	Subgroup	N	β (95% CI)	P for interaction
Child age	<4	4,484	-0.17 (-0.20, -0.14)	0.091
	≥4 to <5	6,797	-0.14 (-0.17, -0.12)	
	≥5	10,085	-0.18 (-0.20, -0.16)	
Child gender	Girls	10,304	-0.17 (-0.19, -0.15)	0.655
	Boys	11,062	-0.16 (-0.18, -0.15)	
Preterm birth	No	20,383	-0.17 (-0.18, -0.15)	0.117
	Yes	983	-0.20 (-0.27, -0.14)	
Respondent role	Father	5,108	-0.14 (-0.16, -0.11)	0.012
	Mother	16,258	-0.18 (-0.19, -0.16)	
Respondent age	<40	18,824	-0.17 (-0.18, -0.15)	0.366
	≥40	2,542	-0.16 (-0.19, -0.13)	
Maternal smoking	No	21,165	-0.17 (-0.18, -0.15)	0.761
	Yes	201	-0.13 (-0.30, 0.03)	
Paternal smoking	No	8,674	-0.17 (-0.19, -0.15)	0.986
	Yes	12,692	-0.17 (-0.18, -0.15)	
Maternal alcohol use	No	20,558	-0.17 (-0.18, -0.15)	0.834
	Yes	808	-0.15 (-0.22, -0.07)	
Paternal alcohol use	No	11,751	-0.16 (-0.18, -0.15)	0.367
	Yes	9,615	-0.17 (-0.19, -0.15)	

Models adjusted for child age, child sex, preterm birth, parental smoking and alcohol use, respondent role, and respondent age. β (95% CI) per 1-SD increase in family SES. Interaction P-values were derived from Wald tests (binary strata) or Type 3 ANOVA (multicategory strata).

### 3.5 Sensitivity analyses

Multiple imputation for missing data yielded results virtually identical to the complete-case analysis (**Supplementary Table 2**). Model diagnostics showed acceptable discrimination for the logistic model (AUC = 0.674) and excellent calibration (Brier score = 0.0051) (**Supplementary Figure 2**). Residual plots, including Q-Q plots and residual-vs-fitted plots, revealed slight deviations from normality and homoscedasticity (**Supplementary Figures 3**, **4**), which were adequately addressed by the cluster-robust standard errors. Taken together, these sensitivity analyses confirm that our findings are robust to missing data, model specification, and minor violations of linear regression assumptions.

## 4 Discussion

In this large cross-sectional study of 21,366 preschool children from western China, we observed that higher family SES was associated with lower ADHD symptom levels, with a dose-response relationship across SES tertiles. The association remained robust after adjusting for a range of covariates and across examined subgroups. These findings are consistent with previous research documenting socioeconomic disparities in child mental health and extend the evidence to the understudied western China region.

Consistent with our findings, research (30) reported that SES was a significant predictor of neurodevelopmental disorders, including ADHD, in a Spanish school-based sample of 6,834 children. In a sample of socioeconomically disadvantaged preschoolers, ADHD behaviors were uniquely associated with poorer school readiness outcomes across multiple domains (31). In a Chinese sample of 2,593 school-age children, higher SES buffered the associations between loneliness and depressive symptoms, highlighting the protective role of socioeconomic resources in child mental health (32).

The mechanisms underlying the observed SES-ADHD association cannot be determined from our cross-sectional data, but previous studies offer plausible pathways. A systematic review found that exposure to low SES was associated with alterations in brain structure, function, and connectivity in ADHD (33). Using Mendelian randomization, Xie et al. (34) showed that SES may influence ADHD through brain structural changes, with the surface area of the insula identified as a partial mediator. Thorsheim et al. (35) suggested that elevated SES and positive parenting may be associated with reduced ADHD symptom severity, with maternal genetic factors potentially influencing these effects. Additionally, Ahlberg et al. (36) found that the association between ADHD and shorter height was partly explained by low SES, along with prenatal factors and psychiatric comorbidity.

Socioeconomic differences may also extend to diagnosis, treatment, and longer-term outcomes. Research (37) found that children with ADHD from lower SES families in Australia had significantly lower medication coverage. In a study of adolescent bipolar disorder, low SES was associated with undertreatment of ADHD (38). A systematic review (39) identified childhood SES as a potential predictor of adult psychiatric outcomes in individuals with childhood ADHD. Parents of children with ADHD have also been found to have lower educational attainment and higher unemployment rates (40). Research (41) examined SES as one of several risk factors for discontinuation of ADHD medication. Together, these observations suggest that low-SES families may face both higher ADHD symptom levels and potentially more limited access to consistent treatment, though longitudinal studies are needed to confirm this pattern.

Our findings also contribute to understanding how SES may relate to other risk factors. Several studies have reported that associations between various ADHD-related factors and outcomes persist after adjusting for SES. For instance, the links between ADHD and short stature (42), the association between hearing impairment and severe ADHD (43), and the relationship between prenatal maternal smoking and childhood ADHD (44) all remained significant after SES adjustment, suggesting that these factors operate through pathways at least partially independent of socioeconomic conditions. Conversely, lower guardian SES and greater ADHD severity jointly increased the likelihood of subsequent substance use in adolescents with Tourette syndrome (45), indicating that socioeconomic disadvantage may compound vulnerability when other risk factors are present. The association between ADHD symptom severity and functional impairment appears to be similar across SES levels (46). Among adults with ADHD, low income was associated with comorbid generalized anxiety disorder (47), while in children with neurodevelopmental conditions, higher SES was linked to fewer co-occurring symptoms (48). Notably, lower SES was associated with lower rates of reported concussion, but this association did not differ by ADHD status (49), suggesting that SES-ADHD interactions may be outcome-specific. Collectively, these findings suggest that the SES-ADHD relationship is complex, with SES potentially interacting with, but not fully accounting for, a range of genetic, environmental, and clinical factors. Our stratified analyses, which showed no significant effect modification by most covariates, are consistent with this complexity, suggesting that while SES exerts a consistent protective effect across subgroups, it does not operate in isolation from other risk factors.

### 4.1 Strengths and limitations

Several strengths of this study warrant mention, including the large sample size, stratified cluster sampling design, comprehensive SES index incorporating both parents’ information, and the use of cluster-robust standard errors. Several limitations should also be acknowledged. First, the cross-sectional design precludes causal inference. We cannot determine the direction of the observed association—whether low SES leads to higher ADHD symptoms, whether child ADHD symptoms contribute to downward socioeconomic mobility, or whether unmeasured factors account for the relationship. Longitudinal studies are needed to establish temporality. Second, ADHD symptoms were assessed using the SDQ hyperactivity/inattention subscale rather than clinical diagnostic interviews. Although we used both the continuous score (as the primary outcome) and a binary indicator (score >6) to capture different aspects of symptom distribution, the SDQ remains a screening instrument and does not equate to a clinical diagnosis. Third, all information was parent-reported, which may introduce reporting bias. Parents experiencing greater stress may systematically over-report child behavioral difficulties, potentially inflating the observed associations. Fourth, we did not collect data on several potentially important confounders, including parental history of ADHD, maternal perinatal health or depression, and quality of the home learning environment. These unmeasured factors may have contributed to residual confounding. Additionally, the sample was restricted to children attending public kindergartens in one western Chinese city, limiting generalizability to children in private kindergartens, those not enrolled in preschool, or those in other regions. Despite these limitations, our findings add to the evidence that socioeconomic disadvantage is associated with higher ADHD symptom levels in preschool children in western China, suggesting that low-SES families may benefit from early intervention strategies, though further research is needed to establish causality.

## 5 Conclusion

In summary, this large cross-sectional study of 21,366 preschool children from western China provides evidence that higher family SES is associated with lower ADHD symptom levels, with a clear dose-response relationship. The association remained robust across subgroups and after adjusting for a wide range of covariates. While the cross-sectional design precludes causal conclusions, our findings extend the existing literature to the understudied western China region and highlight the importance of socioeconomic resources in early childhood mental health. Future longitudinal studies are warranted to elucidate the causal mechanisms underlying this association and to identify specific modifiable pathways. From a public health perspective, these findings may inform the development of early intervention strategies targeting low-SES families with preschool children to reduce the burden of ADHD symptoms and promote healthier developmental trajectories.

## Data Availability

This data set is still being used for analysis. Please contact the corresponding author regarding access to the full dataset. Requests to access the datasets should be directed to Yan Wang, 13630226562@163.com.
