# Supplementary material for: Family socioeconomic status and attention-deficit/hyperactivity disorder symptoms in preschool children: a cross-sectional study of 21,366 parent–child dyads in Yan’an, western China
Source: Front Psychiatry. 2026 Sep 28;17:1943855. doi: 10.3389/fpsyt.2026.1943855 (PMC13655008; doi:10.3389/fpsyt.2026.1943855)
Supplement: Supplementary file 1 [file DataSheet1.zip › Supplementary Table S1-S2.docx]

**Supplementary Table S1. Variance inflation factors for predictors in the fully adjusted linear regression model**

| **Variable** | **VIF** |
| --- | --- |
| SES | 1.05 |
| Child age | 1.03 |
| Child gender | 1.00 |
| Preterm birth | 1.00 |
| Respondent role (mother) | 1.07 |
| Respondent age | 1.06 |
| Maternal smoking | 1.13 |
| Paternal smoking | 1.20 |
| Maternal alcohol use | 1.13 |
| Paternal alcohol use | 1.20 |

**Supplementary Table S2. Sensitivity analysis using multiple imputation for missing data**

| **Outcome** | **Complete case**  **analysis (n=21,366)** | **Multiple imputation (n=25,017)** | **P value** |
| --- | --- | --- | --- |
| ADHD symptom score (continuous)  (β, 95% CI) | -0.17 (-0.18, -0.15) | -0.17 (-0.19, -0.16) | <0.001 |
| Elevated ADHD symptoms (binary) (OR, 95% CI) | 0.73 (0.64, 0.84) | 0.74 (0.66, 0.84) | <0.001 |

**Supplementary Figure legends**

**Supplementary Figure S1.** Restricted cubic spline (4 knots) of the association between family SES and child ADHD symptoms. Adjusted for all covariates. The solid line represents predicted ADHD symptom scores across SES levels; shaded bands indicate 95% confidence intervals. All other covariates were fixed at reference levels for categorical variables and at medians for continuous variables.

**Supplementary Figure S2.** ROC curve of the fully adjusted logistic regression model for binary ADHD symptoms (score >6). AUC = 0.674, indicating acceptable discriminative ability.

**Supplementary Figure S3.** Normal Q-Q plot of residuals from the fully adjusted linear regression model. Points generally follow the diagonal line, with minor deviations at the tails, suggesting approximately normal residuals.

**Supplementary Figure S4.** Residuals vs fitted plot from the fully adjusted linear regression model. Points are randomly distributed around the horizontal line at zero, with no obvious patterns, supporting the assumptions of linearity and homoscedasticity.

All models adjusted for child age, child sex, preterm birth, maternal smoking, paternal smoking, maternal alcohol use, paternal alcohol use, respondent role, and respondent age.
